# The impact of positive end-expiratory pressure on right ventricular function in patients with moderate-to-severe ARDS: a prospective paired-design study

**DOI:** 10.3389/fmed.2024.1424090

**Published:** 2024-07-02

**Authors:** Luo Xingzheng, Gu Weiguang, Ye Quanqiu, Zhou Huifen, Zheng Zijun, Zou Qiming, Yuan Suhua, Zhang Fu, Jian Zhigang

**Affiliations:** ^1^Department of Critical Care Medicine, Xiaolan People’s Hospital of Zhongshan, Zhongshan, Guangdong, China; ^2^Department of Medical Records, Xiaolan People’s Hospital of Zhongshan, Zhongshan, Guangdong, China; ^3^Department of Ultrasound, Xiaolan People’s Hospital of Zhongshan, Zhongshan, Guangdong, China

**Keywords:** positive end-expiratory pressure, right ventricular function, acute cor pulmonale, ARDS, hemodynamics

## Abstract

**Objective:**

To determine the effects of varying positive end-expiratory pressures (PEEPs) on right ventricular function, hemodynamics, oxygenation, and the incidence of acute cor pulmonale (ACP) in patients with moderate-to-severe acute respiratory distress syndrome (ARDS).

**Methods:**

This prospective paired-design study involved patients with moderate-to-severe ARDS in the ICU. Participants received lung-protective ventilation and hemodynamic monitoring. During the study, mechanical ventilation was administered with PEEPs of 5 cmH2O, 10 cmH2O, and 15 cmH2O, while maintaining an end-inspiratory plateau pressure ≤ 30 cmH2O. Various assessments, including transthoracic echocardiography, cardiac output measurement, and blood gas analysis, were conducted at baseline and after 1 h of ventilation at each PEEP. Subsequently, variations in ventilation oxygenation, echocardiographic parameters, and hemodynamic indicators under different PEEPs were analyzed to explore the potential effects of PEEP on right ventricular function and hemodynamics, as well as the incidence of ACP.

**Results:**

A total of 317 ARDS patients were screened. Among them, 104 met the diagnostic criteria for moderate-to-severe ARDS, and 52 completed the study. The baseline PEEP of these 52 participants, acquired before commencement, was 11.5 ± 1.7 cmH2O, and the incidence of ACP was 25.0% (13/52). Intensive care unit mortality, overall hospital mortality, and 28-day mortality rates were 19.2% (10/52), 21.2% (11/52), and 32.7% (17/52), respectively. During the study, ACP incidences at PEEPs of 5 cmH2O, 10 cmH2O, and 15 cmH2O were 17.3% (9/52), 21.2% (11/52), and 38.5% (20/52), respectively. Meanwhile, the PaO2/FiO2 ratio improved with increasing PEEP, reaching 162.0 (140.9, 174.0), 171.0 (144.0, 182.0), and 176.5 (151.0, 196) mmHg at PEEPs of 5 cmH2O, 10 cmH2O, and 15 cmH2O, respectively. In addition, higher PEEPs were associated with a slight increase in PaCO_2_, showing statistically significant differences compared to moderate and low PEEPs. Compared to a PEEP of 5 cmH2O or 10 cmH2O, right ventricular function exhibited substantial changes at 15 cmH2O PEEP, manifested as increased pulmonary artery systolic pressure, enlarged right ventricular end-diastolic area, and decreased tricuspid annular plane systolic excursion, all with significant differences. Conversely, variations in left ventricular end-diastolic area and ejection fraction were not statistically significant. In terms of hemodynamics, increasing PEEP resulted in a decline in cardiac index (CI), with statistically significant differences between different PEEPs. Specifically, compared to the value at a PEEP of 5 cmH2O, the CI at a PEEP of 15 cmH2O decreased by 14.3% (2.63 [2.20, 2.95] vs. 3.07 [2.69, 3.67], *p* < 0.001). The decline in the stroke volume index with PEEP was more obvious (22.1 [18.4, 27.1] vs. 27.0 [24.2, 33.0], *p* < 0.001), reaching 18.1%. Additionally, both end-diastolic volume index and extravascular lung water index decreased significantly with increasing PEEP, while the pulmonary vascular permeability index remained unaffected.

**Conclusion:**

Different PEEPs can affect the incidence of ACP in patients with moderate-to-severe ARDS. High PEEP improves oxygenation and reduces extravascular lung water without significantly affecting the pulmonary vascular permeability index and left ventricular systolic function. Nevertheless, it can cause right ventricular dilation, as well as substantial declines in right ventricular systolic function and CI, thereby causing ACP.

## 1 Introduction

Acute respiratory distress syndrome (ARDS) is a common clinical syndrome in the intensive care unit (ICU) (1, 2). ARDS is associated with high morbidity and mortality, with mortality rates for moderate and severe ARDS reaching 32 and 45%, respectively (3). In patients with severe acute respiratory failure, elevated pulmonary vascular resistance can increase right ventricular afterload, leading to right ventricular dysfunction and, in severe cases, acute cor pulmonale (ACP) and refractory circulatory failure (4). Mechanical ventilation is a standard respiratory support method for ARDS patients. However, inappropriate mechanical ventilation settings can increase pulmonary vascular resistance and pulmonary arterial pressure, causing right ventricular dysfunction or ACP (5), thereby prolonging mechanical ventilation and worsening prognosis.

The severity of ARDS and the parameters of mechanical ventilation are key factors in the incidence of right ventricular dysfunction and ACP (6). For ARDS patients, appropriate positive end-expiratory pressure (PEEP) can reopen collapsed alveoli, reduce pulmonary arterial resistance, and decrease right ventricular afterload. Both excessively low and high PEEPs can increase pulmonary vascular resistance, impair pulmonary circulation, and compromise right ventricular function, ultimately leading to circulatory failure (7). Nevertheless, the effects of different PEEPs on right ventricular function in ARDS patients and their relationship with ACP are not well understood and require further elaboration.

Therefore, in this study, mechanical ventilation with varying PEEPs was performed on patients with moderate-to-severe ARDS. Data on oxygenation parameters, cardiac function, and hemodynamic indicators were subsequently collected to evaluate the effects of different PEEPs on right ventricular function and hemodynamics in these patients. Additionally, whether different PEEPs influenced the incidence of ACP was investigated.

## 2 Methods

This study is a single-center, prospective, paired-design study. During the study, mechanical ventilation was administered sequentially with PEEPs of 5 cmH2O, 10 cmH2O, and 15 cmH2O to patients diagnosed with moderate-to-severe ARDS. Data on their ventilation oxygenation status, cardiac function, and hemodynamic parameters were collected during this process.

The study protocol was approved by the Ethics Review Committee of Xiaolan People’s Hospital of Zhongshan (approval number: 200421113453850). All research activities complied with the Declaration of Helsinki and its amendments, as well as the clinical research regulations of Xiaolan People’s Hospital of Zhongshan.

### 2.1 Study subjects

Continuous data collection was conducted on ARDS patients who were hospitalized in the ICU of Xiaolan People’s Hospital of Zhongshan and received mechanical ventilation from July 1, 2020, to October 31, 2022. The inclusion criteria were (1) endotracheal intubation via the mouth or nose; (2) clinical diagnosis of ARDS; and (3) PaO_2_/FiO_2_ ratio ≤ 200 mmHg. The exclusion criteria were (1) age < 18 years; (2) chronic cor pulmonale, diagnosed based on the “Guidelines for Primary Care of Chronic Cor Pulmonale (2018)” proposed by the Chinese Medical Association (8); (3) right ventricular myocardial infarction; (4) acute pulmonary embolism; (5) pregnant or breastfeeding women; (6) hemodynamic instability (requiring more than 1 μg/kg/min of epinephrine or norepinephrine within the past 2 h, progressively increasing maintenance dose or dependence on fluid boluses); (7) pneumothorax or barotrauma; and (8) and intracranial hypertension.

Patients meeting all the inclusion criteria and none of the exclusion criteria were considered potential study subjects. These patients entered a screening phase where they received ventilation according to the ARDS Net protocol for 1 h, and PEEP and FiO2 were set according to the PEEP-FiO2 table according to the Lower PEEP/Higher FiO2 strategy. If blood gas analysis indicated a PaO_2_/FiO_2_ ratio ≤ 200 mmHg, the patient was confirmed as eligible subjects. For all of these patients, informed consent was obtained from their legal representatives, and consent forms were signed. At this point, the study subjects were officially enrolled and became participants. Participants received adequate sedation, analgesia, and short-term neuromuscular blockade. Patients with fluid deficits received preemptive fluid resuscitation. Subsequently, in the experimental phase, mechanical ventilation with PEEPs of 5 cmH2O, 10 cmH2O, and 15 cmH2O was administered sequentially for 1 h each, during which FiO_2_ was adjusted to maintain a target finger oxygen saturation of 88–95%. After 1 h of ventilation at each PEEP, arterial blood gas analysis, transthoracic echocardiography, and cardiac output measurement via thermodilution were performed sequentially.

### 2.2 Definitions and criteria

The diagnostic criteria for ARDS were based on the Berlin definition published in 2012 in the Journal of the American Medical Association (3). ARDS was diagnosed when all the following conditions were met. (1) Timing: within 1 week of a known clinical onset or new or worsening respiratory symptoms. (2) Chest Imaging: X-ray or computed tomography scan indicating bilateral opacities not fully explained by effusions, lobar/lung collapse, or nodules. (3) Origin of edema: respiratory failure not fully explained by cardiac failure or fluid overload. If no risk factors are present, objective assessment (e.g., echocardiography) is required to exclude hydrostatic edema. (4) Oxygenation: the PaO_2_/FiO_2_ ratio is ≤300 mmHg under a PEEP or continuous positive airway pressure ≥ 5 cm H2O. For patients meeting the above diagnostic criteria, the severity of ARDS was classified based on the PaO_2_/FiO_2_ ratio. Specifically, 200 mmHg < PaO_2_/FiO_2_ ratio ≤ 300 mmHg was classified as mild ARDS, 100 mmHg < PaO_2_/FiO_2_ ratio ≤ 200 mmHg as moderate ARDS, and PaO_2_/FiO_2_ ratio ≤ 100 mmHg as severe ARDS.

In this study, chronic underlying conditions included chronic obstructive pulmonary disease; chronic heart failure (New York Heart Association Class III-IV); metastatic cancer (confirmed by surgery or imaging); liver cirrhosis; chronic renal failure (chronic renal insufficiency requiring maintenance dialysis or with a serum creatinine >300 μmol/L); immunosuppression (having received corticosteroid therapy with a daily prednisone equivalent ≥0.3 mg/kg for at least 1 month within the past 6 months, severe malnutrition, congenital humoral or cellular immunity deficiency, or having received chemotherapy/radiotherapy within the past 6 months); and diabetes.

The diagnosis of right ventricular dysfunction was based on the ratio between the right ventricular end-diastolic area (RVEDA) and the left ventricular end-diastolic area (LVEDA). An RVEDA/LVEDA ratio between 0.6 and 1 indicated mild right ventricular dilation, while a ratio > 1 suggested severe right ventricular dilation. Additionally, a tricuspid annular plane systolic excursion (TAPSE) < 16 mm indicated right ventricular systolic dysfunction (9).

The diagnostic criteria for ACP included an RVEDA/LVEDA >0.6 and the presence of paradoxical septal motion (10).

### 2.3 Measurement and calculation of indicators

Right ventricular function indicators were obtained using transthoracic echocardiography with a SonoSite X-Porte ultrasound machine and a 5 Hz phased-array probe. All operators completed advanced cardiac ultrasound and basic critical care ultrasound training through the Critical Care Ultrasound Study Group. Echocardiographic measurements were acquired at the end of expiration. The RVEDA/LVEDA ratio was measured in the apical four-chamber view using the area-length method in a single plane. The TAPSE was obtained via an M-mode line through the tricuspid valve’s lateral annulus, while the pulmonary artery systolic pressure (PASP) was calculated from the velocity of tricuspid regurgitation. The diameter of the inferior vena cava (IVC) was measured in the subxiphoid view with the probe longitudinally aligned to the post-hepatic IVC. Measurements were taken 2 cm from the right atrium entrance, freezing the ultrasound image at the end of inspiration and expiration to determine the maximum (IVCmax) and minimum (IVCmin) diameters. These values were subsequently used to calculate the distensibility index.


$$\begin{array}{l} \mathrm{Distensibility Index of the}\;\mathrm{IVC}\;\left(\mathrm{DIVC}\right)\\ \;\;\;\;\;\;\;\;\;\;\;\;\;\;\;\;=\left(\mathrm{IVCmax}-\mathrm{IVCmin}\right)/\mathrm{IVCmin}\times 100\% \end{array}$$

During cardiac output measurement, all participants were equipped with the Edwards EV1000 monitoring kit for continuous hemodynamic monitoring. To ensure the stability and reliability of the data, all operators underwent standardized training and assessment procedures. For transpulmonary thermodilution measurements, a bolus of ice-cold saline (temperature below 8°C) with a volume of 15 mL was used. Using this approach, we measured cardiac output, global end-diastolic volume, and intrathoracic thermal volume. These measurements were then used to derive the extravascular lung water index (EVLWI), cardiac index (CI), global end-diastolic volume index (GEDVI), stroke volume index (SVI), systemic vascular resistance index (SVRI), and pulmonary vascular permeability index (PVPI).

The length of hospital stay was calculated using the time of admission to the ICU as the starting point (Timepoint 0). Follow-up continued for 28 days. On this basis, the length of ICU stay, total hospital stay, and follow-up days were calculated.

### 2.4 Clinical data collection

During the screening period, relevant information about the subjects was collected, including demographic data. Furthermore, during both the screening and intervention periods, several immediate vital signs were recorded, including heart rate, mean arterial pressure, systolic blood pressure, central venous pressure, respiratory rate, Acute Physiology and Chronic Health Evaluation II (APACHE-II) score, transthoracic echocardiography results (RVEDA/LVEDA ratio, TAPSE, IVCmax, IVCmin, DIVC, and PASP), blood gas analysis results (pH, PaO_2_, PaCO_2_, lactate, and PaO_2/_FiO_2_ ratio), and hemodynamic data (CI, SVI, SVRI, GEDVI, EVLWI, and PVPI). For all study subjects, the following observational indicators were also collected, namely the incidence of ACP, acute kidney injury (AKI), and acute renal failure (ARF); length of ICU stay; total hospital stay; ICU mortality rate; hospital mortality rate; and 28-day mortality rate.

### 2.5 Sample size estimation

A previous large prospective study showed that the incidence of ACP in moderate-to-severe ARDS was approximately 22% (11). Therefore, an ACP incidence of 30% with high PEEP and 15% with low PEEP was assumed in the study. Using Stata/MP17.0 statistical software and McNemar’s test for paired sample proportions, with a significance level of α = 0.05 and a power of 80%, the sample size was initially estimated to be 62 cases. Considering a 10% rate of missing or excluded cases, the final estimated sample size was 69 cases. During the actual trial, a significantly escalated incidence of ACP with high PEEP was observed compared to low PEEP. Due to this significant difference, the study was terminated early after completing the trial with 52 patients.

### 2.6 Statistical analysis

Data processing and statistical analysis were performed using Stata/MP17.0 software, with GraphPad Prism 9.0 used for chart creation. The normality of continuous variables was tested using skewness and kurtosis. Categorical variables are presented as frequencies and percentages, while continuous variables are presented as mean and standard deviation or median and interquartile range, depending on their distribution normality. The incidence of ACP, right ventricular function, hemodynamic parameters, and oxygenation parameters between different PEEPs were compared based on the nature of the variables. For categorical variables, the McNemar chi-square test or Fisher’s exact test was utilized. For continuous variables, the paired *t*-test or Wilcoxon signed-rank test was employed. To further identify independent risk factors associated with ACP, backward stepwise logistic regression analysis was performed on potentially significant risk factors (*p* < 0.20 in univariate analysis). Meanwhile, the relationship between ACP and outcomes (e.g., length of hospital stay, mortality) was analyzed using chi-square tests, independent *t*-tests, or Mann–Whitney U tests. All hypothesis tests were two-sided, and a *p*-value <0.05 was considered statistically significant.

## 3 Results

### 3.1 Patient basic information

**Figure 1:**
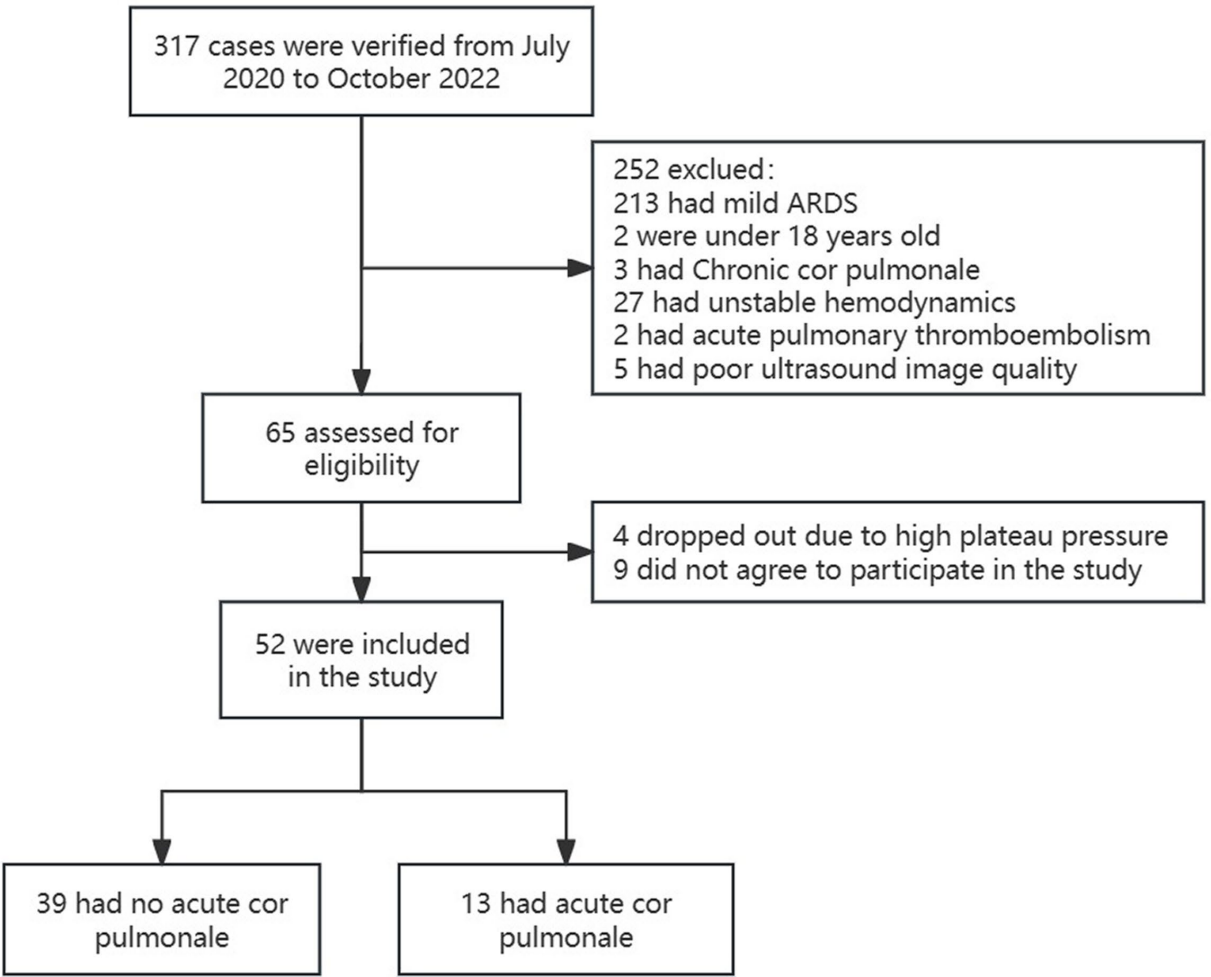
Flowchart of patients in the study.

During the study period, the information of a total of 317 ARDS patients was consecutively collected at Xiaolan People’s Hospital of Zhongshan. During the same period, 3,208 critically ill patients were treated at the hospital, indicating an ARDS incidence rate of 9.9%. Exclusions included 213 patients with mild ARDS, 18 patients under 18 years of age, three with chronic cor pulmonale, two with acute pulmonary thromboembolism, five with poor ultrasound image quality, and 27 with severe hemodynamic instability. This left 65 patients who met the study criteria and entered the intervention period. During this period, four patients with persistent plateau pressure above 30 cmH2O and nine who did not provide informed consent were excluded, resulting in 52 ARDS patients included in the study (Figure 1).

The median age of the 52 patients was 56.5 years (46.0, 71.5), with 36 males (69.2%) and 16 females (30.8%). The most common cause of ARDS was pneumonia, accounting for 67.3% (35/52) of the cases, followed by sepsis from other sites at 11.5% (6/52). The median APACHE-II score was 20 (17.5, 22.5). Prior to the intervention, the incidence of ACP was 25.0% (13/52), and the PEEP used was 11.5 ± 1.7 cmH2O. Chronic underlying diseases were present in 59.6% (31/52) of the patients, with diabetes being the most common at 26.9%. The median lengths of ICU stay and total hospital stay for the 52 patients were 12.4 days (6.5, 16.0) and 12.7 days (12.0, 25.0), respectively (Table 1). The ICU mortality rate, 28-day mortality rate, and hospital mortality rate were 19.2% (10/52), 32.7% (17/52), and 21.2% (11/52), respectively (Table 2).

**Table 1:** Clinical characteristics of patients in this study, as well as oxygenation, cardiac ultrasound, and hemodynamic data.

General characteristics	Total (*N* = 52)	ACP group (*N* = 13) 25.0%	non-ACP group (*N* = 39) 75.0%	t/z/χ^2^	*p*
Sex, male, *n* (%)	36 (69.2)	9 (69.2)	27 (69.2)	0.000	1.000
Age, years, (median [IQR])	56.5 [46.0, 71.5]	54.0 [49.0, 63.0]	64.0 [41.0, 72.0]	0.867	0.386
BMI, kg/m^2^, (*x-±s*)	24.1 ± 3.74	25.9 ± 4.4	23.5 ± 3.3	−2.026	0.048
BMI > 25, *n* (%)	19 (36.5)	8 (61.5)	11 (28.2)	_	0.047
BSA, m^2^, (*x-±s*)	1.66 ± 0.16	1.71 ± 0.14	1.64 ± 0.17	−1.499	0.140
Etiology of ARDS, *n* (%)					
Pneumonia	35 (67.3)	11 (84.6)	24 (61.5)	_	0.178
Other sepsis	6 (11.5)	1 (7.7)	2 (12.8)	_	1.000
Pancreatitis	3 (5.8)	1 (7.7)	2 (5.1)	_	1.000
Gas inhalation	3 (5.7)	0	3 (7.7)	_	0.564
Burn	3 (5.7)	0	3 (7.7)	_	0.564
Trauma	2 (3.8)	0	2 (5.1)	_	1.000
Chronic underlying disease, *n* (%)	31 (59.6)	10 (76.9)	21 (53.8)	2.157	0.142
Chronic heart failure	3 (5.8)	1 (7.7)	2 (5.1)	_	1.000
COPD	11 (21.2)	4 (30.8)	5 (12.8)	_	0.203
Cirrhosis	3 (5.8)	1 (7.7)	2 (5.1)	_	1.000
Chronic renal failure	3 (5.8)	2 (15.4)	1 (2.6)	_	0.151
Metastatic cancer	3 (5.8)	1 (7.7)	2 (5.1)	_	1.000
Immunosuppression	4 (7.7)	2 (15.4)	2 (5.1)	_	0.257
Diabetes	14 (26.9)	2 (15.4)	12 (30.8)	_	0.145
APCHE-II, (median [IQR])	20.0 [17.5, 22.5]	20.0 [18.0, 22.0]	20.0 [17.0, 23.0]	−0.531	0.596
Vt，ml/kg/pbw，(*x-±s*)	6.6 ± 0.5	6.6 ± 0.6	6.6 ± 0.5	0.226	0.822
Vf, bpm, (x̅±s)	19.4 ± 2.6	18.9 ± 2.9	19.5 ± 2.5	0.703	0.485
MinVent, L/min, (x̅±s)	7.78 ± 1.17	7.62 ± 1.41	7.83 ± 1.09	0.566	0.574
PEEP, cmH2O, (*x-±s*)	11.8 ± 1.8	13.2 ± 1.1	11.3 ± 1.8	−3.470	0.001
Pplateau, cmH2O, (x̅±s)	26.4 ± 2.1	28.2 ± 2.0	25.8 ± 1.8	−3.946	0.002
Driving Pressure, cmH2O, (x̅±s)	14.7 ± 2.5	15.0 ± 2.2	14.5 ± 2.6	−0.579	0.565
pH, (*x-±s*)	7.39 ± 0.07	7.36 ± 0.06	7.40 ± 0.06	1.946	0.057
PaO_2_, mmHg, (median [IQR])	86.0 [77.0, 95.0]	85.0 [77.0, 88.0]	86.0 [76.0, 96.0]	0.381	0.703
PaCO_2_, mmHg, (*x-±s*)	40.3 ± 4.9	43.9 ± 5.3	39.2 ± 4.2	−3.262	0.002
PaO_2_/FiO_2_ ratio, mmHg, (*x-±s*)	155.7 ± 28.1	150.9 ± 24.1	157.3 ± 29.5	0.696	0.490
RVEDA, cm^2^, (*x-±s*)	19.3 ± 2.3	20.7 ± 2.0	18.9 ± 2.2	−2.565	0.013
LVEDA, cm^2^, (*x-±s*)	33.3 ± 4.3	32.2 ± 4.2	33.7 ± 4.3	1.081	0.285
RVEDA/LVEDA, (*x-±s*)	0.59 ± 0.07	0.65 ± 0.1	0.57 ± 0.1	−3.741	<0.001
TAPSE, mm, (median [IQR])	16.0 [14.0, 18.0]	14.0 [13.0, 14.0]	17.0 [16.0, 18.0]	4.897	<0.001
IVCmax, mm, (median [IQR])	19.8 [18.8, 23.2]	21.3 [18.9, 23.4]	19.8 [18.8, 23.2]	−1.334	0.182
IVCmin, mm, (median [IQR])	16.6 [14.3, 19.8]	19.8 [15.6, 21.6]	15.6 [13.7, 17.8]	−1.708	0.088
DIVC, %, (median [IQR])	21.2 [16.5, 37.1]	20.5 [12.9, 22.7]	21.2 [17.2, 40.0]	1.406	0.160
PASP, mmHg, (median [IQR])	35.0 [26.0, 43.5]	48.0 [36.0, 55.0]	29.0 [23.0, 42.0]	−3.491	<0.001
LVEF, %, (*x-±s*)	55.3 ± 6.6	54.0[52.0, 58.0]	56.0[52.0, 61.0]	0.834	0.408
CVP, mmHg, (median [IQR])	12.0 [10.0, 13.0]	12.0 [11.0, 13.0]	12.0 [9.0, 13.0]	−1.280	0.200
CI, L/min/m^2^, (median [IQR])	3.02 [2.58, 3.60]	2.59 [2.02, 3.03]	3.10 [2.80, 3.61]	2.187	0.028
SVI, ml/m^2^, (median [IQR])	25.8 [22.6, 31.2]	23.9 [21.8, 27.0]	26.6 [24.0, 31.6]	1.595	0.110
GEDVI, ml/m^2^, (median [IQR])	688.0 [653.0, 733.0]	709.0 [678.0, 738.0]	685.0 [649.0, 732.0]	−0.634	0.526
EVLWI, ml/kg, (median [IQR])	12.6 [11.0, 14.5]	13.8 [11.6, 14.5]	12.6 [11.0, 14.6]	−0.476	0.634
PVPI, (median [IQR])	4.96 [4.30, 5.62]	5.00 [4.79, 5.63]	4.92 [4.28, 5.55]	−0.158	0.874
Vasopressor agents, ug/kg/min					
Noradrenalin, (x̅±s)	0.51 ± 0.18 (*n* = 35)	0.61 ± 0.18 (*n* = 12)	0.46 ± 0.17 (*n* = 23)	−2.400	0.022
Adrenalin, (x̅±s)	0.19 ± 0.08 (*n* = 8)	0.21 ± 0.08 (*n* = 4)	0.16 ± 0.06 (*n* = 4)	−0.985	0.363
Dopamine, (x̅±s)	6.93 ± 2.23 (*n* = 21)	7.60 ± 3.36 (*n* = 5)	6.72 ± 1.84 (*n* = 16)	−0.765	0.454

ACP, acute cor pulmonale; BMI, body mass index; BSA, body surface area; COPD, chronic obstructive pulmonary disease; RVEDA, right ventricular end-diastolic area; LVEDA, left ventricular end-diastolic area; TAPSE, tricuspid annular plane systolic excursion; IVC, inferior vena cava; DIVC, Distensibility index of the IVC; PASP, pulmonary artery systolic pressure; LVEF, left ventricular ejection fractions; CVP, central venous pressure; CI, cardiac index; SVI, stroke volume index; GEDVI, global end-diastolic volume index; EVLWI, extravascular lung water index; PVPI, pulmonary vascular permeability index.

**Table 2:** Factors associated with acute cor pulmonale in patients with ARDS.

Variables	Odds ratio (95% CI) by logistic regression	
	Univariate	Multivariable^a^
BMI > 25 kg/m^2^	4.07 (1.09–15.20), *p* = 0.037	7.28 (0.83–63.56), I/NR
Pneumonia as a cause of ARDS	3.44 (0.67–17.70), *p* = 0.140	4.02 (0.23–69.96), I/NR
Chronic underlying disease	2.86 (0.68–12.00), *p* = 0.152	2.20 (0.27–17.76), I/NR
PEEP >12cmH_2_O	6.53 (1.64–25.93), *p* = 0.008	6.48 (1.54–27.14), *p* = 0.010
pH < 7.35	3.02 (0.67–13.63), *p* = 0.150	0.93 (0.11–7.66), I/NR
PaCO_2_ > 45 mmHg	16.89 (1.68–169.9), *p* = 0.016	6.95 (0.68–71.3), I/NR

ARDS, acute respiratory distress syndrome; CI, confidence interval; BMI, body mass index; I/NR included but not retained by the final model.

a The multivariable model showed a good calibration as assessed by the Pearson goodness of fit test [χ^2^(18 df) = 22.26, *p* = 0.221] and fair discrimination as assessed by the receiver operating characteristics curve [area under the curve (AUC) 0.878].

In the study cohort, there were no statistically significant differences in gender, age, presence of chronic underlying diseases, cause of ARDS, or APACHE-II scores between ARDS patients with ACP and those without ACP. Nevertheless, compared to ARDS patients without ACP, those with ACP exhibited a higher body mass index, higher PEEP, higher arterial PaCO_2_, lower cardiac output, and the need for more vasoactive drugs, all with statistically significant differences. However, no difference was observed in the PaO2/FiO2 ratio (Table 1).

### 3.2 Effects of different PEEPs on cardiac function and hemodynamics in patients with moderate-to-severe ARDS

**Figure 2:**
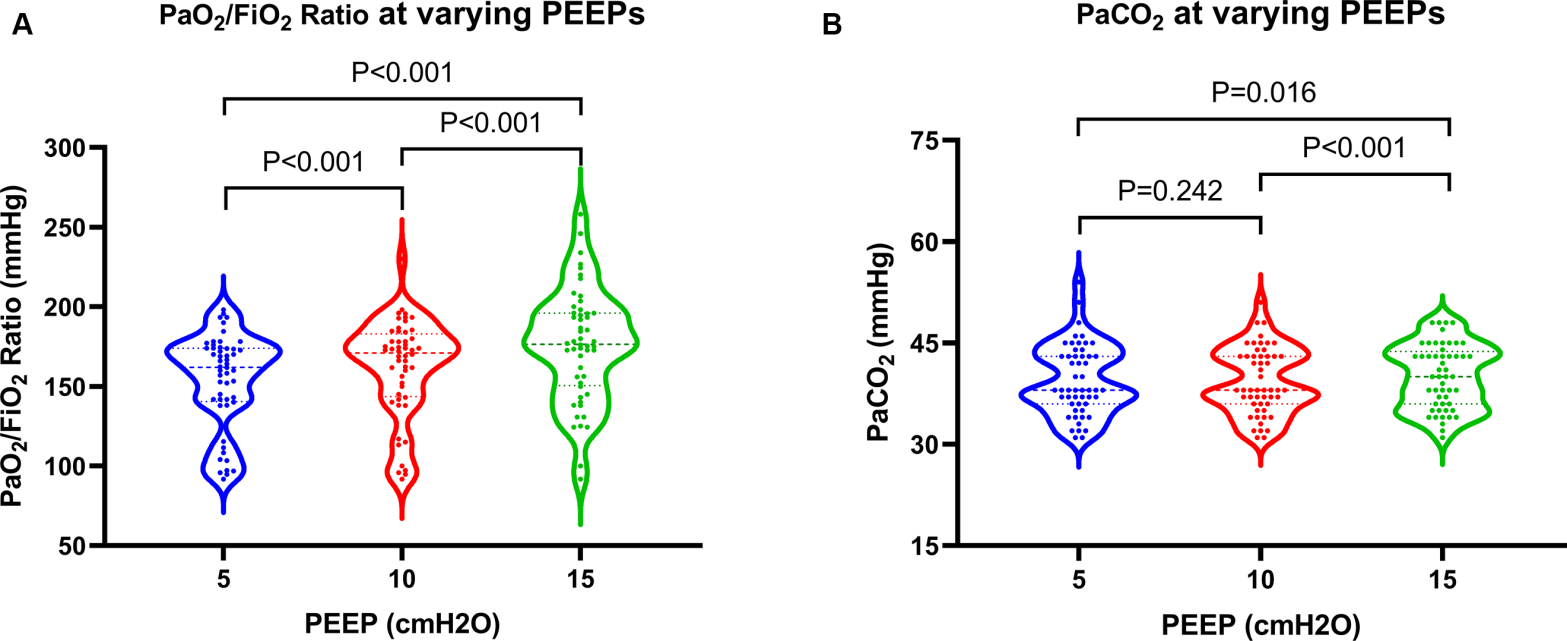
Changes in oxygenation and ventilation in patients at varying PEEPs. PEEP, positive end-expiratory pressure.

All 52 patients underwent cardiac ultrasound and cardiac output measurements at various PEEPs without experiencing pneumothorax or significant hemodynamic deterioration during the trial. The incidence rates of ACP at PEEPs of 5 cmH2O, 10 cmH2O, and 15 cmH2O were 17.3, 21.2, and 38.5%, respectively. There was no statistically significant difference in ACP incidence between PEEPs of 5 cmH2O and 10 cmH2O. However, a statistically significant difference in ACP incidence was observed when comparing a PEEP of 15 cmH2O with values of 5 cmH2O and 10 cmH2O. In addition, a statistically significant increase in the PaO_2_/FiO_2_ ratio was observed with higher PEEP levels. Nonetheless, at a high PEEP, ventilation function slightly declined, leading to a mild elevation in PaCO_2_, which was statistically significant compared to moderate and low PEEPs (Figure 2).

**Figure 3:**
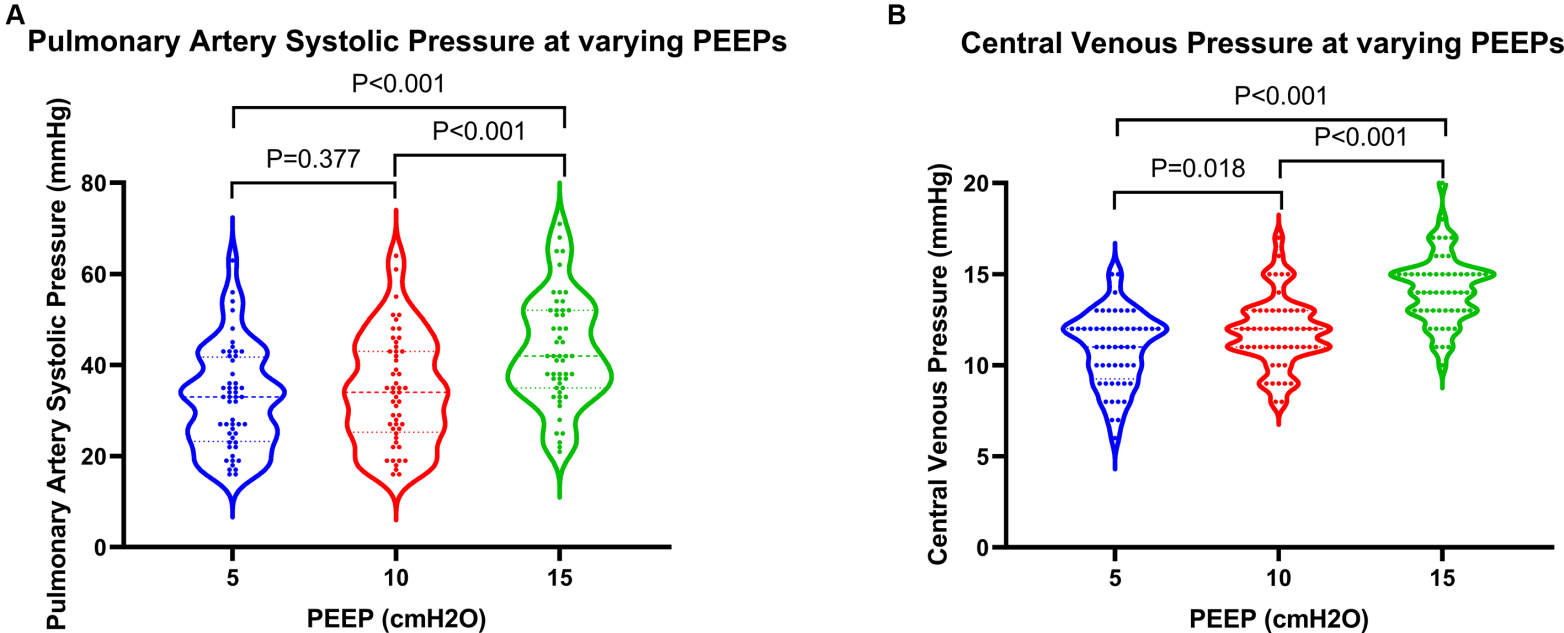
Changes in pressure parameters in patients at varying PEEPs. PEEP, positive end-expiratory pressure.

**Figure 4:**
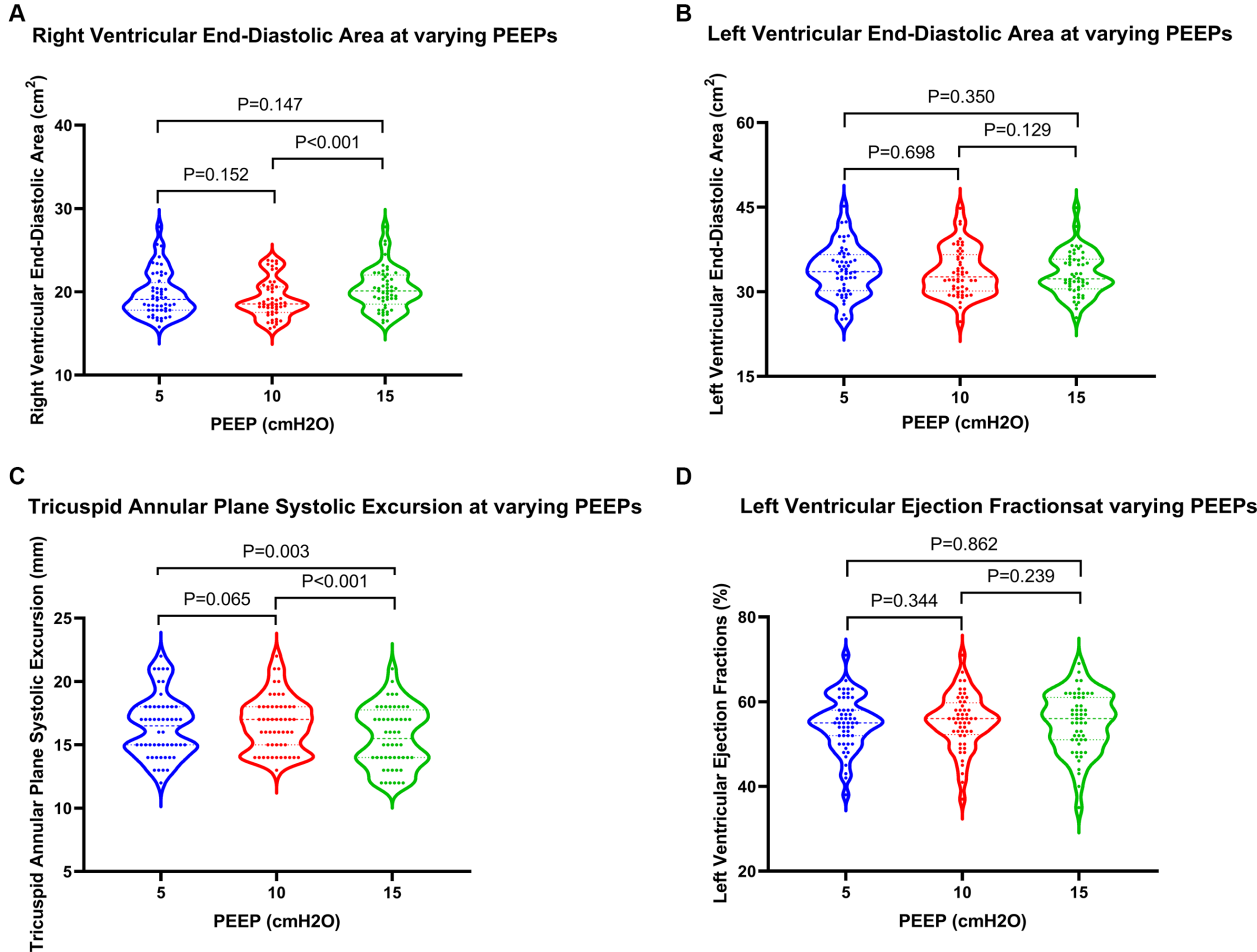
Changes in cardiac function in patients at varying PEEPs. PEEP, positive end-expiratory pressure.

Regarding cardiac function, when PEEP was set at 15 cmH2O, both PASP and CVP demonstrated significant increases, while TAPSE showed a notable decrease (Figure 3). These differences were statistically significant when compared to PEEPs of 5 cmH2O and 10 cmH2O. Furthermore, under a PEEP of 15 cm H2O, RVEDA exhibited considerable enlargement compared to PEEP at 10 cmH2O, with a statistically significant difference. Although there were variations in LVEDA and LVEF under different PEEPs, none of these variances reached statistical significance (Figure 4).

**Figure 5:**
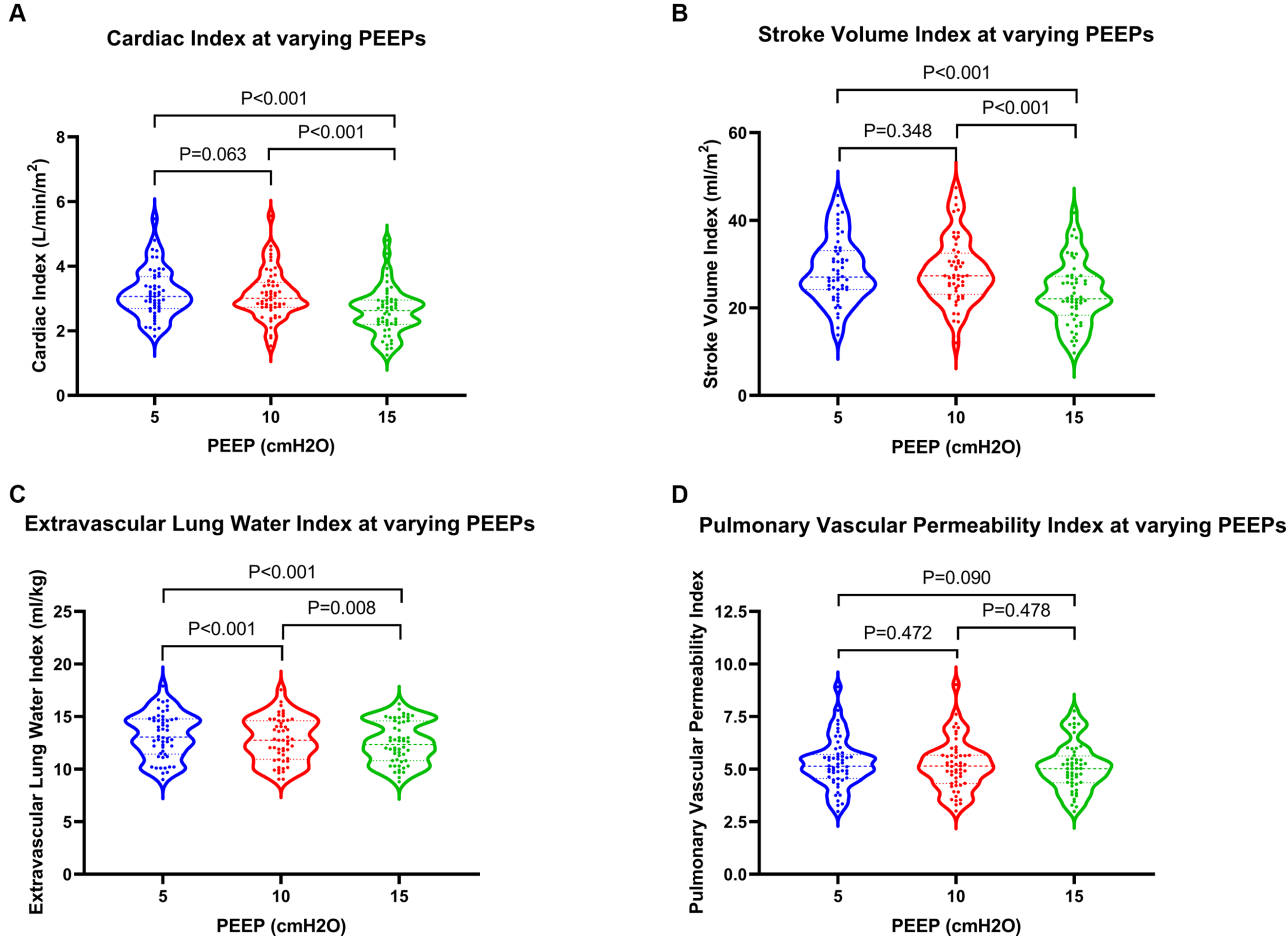
Changes in hemodynamics and extravascular lung water in patients at varying PEEPs. PEEP, positive end-expiratory pressure.

With rising PEEP, patients initially experienced a slight increase followed by a significant decrease in SVI, while CI showed a gradual decline. Comparisons between PEEPs of 10 cmH2O and 5 cmH2O showed no statistically significant differences, whereas comparisons between PEEPs of 15 cmH2O and both 5 cmH2O and 10 cmH2O revealed significant differences. Specifically, when PEEP was set at 15 cmH2O, CI decreased by 14.3% compared to 5 cmH2O (2.63 L/min/m^2^ vs. 3.07 L/min/m^2^), while SVI exhibited a more pronounced decrease of 18.1%. Alternatively, GEDVI and EVLWI gradually decreased with increasing PEEP, with statistically significant differences (*p* values <0.05). Conversely, PVPI did not experience significant changes, with *p* values >0.05 (Figure 5; Table 3).

**Table 3:** Effects of varying PEEPs on cardiac function and hemodynamics.

Parameters	Baseline	PEEP = 5	PEEP = 10	PEEP = 15	t/z/χ^2^ (PEEP 10 vs. 5 cmH2O)	*p* (PEEP 10 vs. 5 cmH2O)	t/z/χ^2^ (PEEP 15 vs. 5 cmH2O)	*p* (PEEP 15 vs. 5 cmH2O)	t/z/χ^2^ (PEEP 15 vs. 10 cmH2O)	*p* (PEEP 15 vs. 10 cmH2O)
ACP, *n* (%)	13 (25.0)	9 (17.3)	11 (21.2)	20 (38.5)	1.000	0.317	11.000	<0.001	14.00	<0.001
PaO_2_, mmHg, (median [IQR])	86.0 [77.0, 95.0]	85.5 [77.0, 94.0]	87.5 [78.0, 98.0]	98.0 [87.0, 106.0]	4.735	<0.001	5.590	<0.001	4.621	<0.001
PaCO_2_, mmHg, (x̅±SD)	40.3 ± 4.9	39.5 ± 5.2	39.3 ± 4.9	40.1 ± 4.7	−1.184	0.242	2.492	0.016	3.860	<0.001
PaO_2_/FiO_2_ ratio, mmHg, (median [IQR])	162.0 [140.0, 174.3]	162.0 [140.9, 174.0]	171.0 [144.0, 182.0]	176.5 [151.0, 196]	4.734	<0.001	5.585	<0.001	4.667	<0.001
RVEDA, cm^2^, (x̅±SD)	19.3 ± 2.3	19.7 ± 2.7	19.1 ± 2.3	20.3 ± 2.5	−1.456	0.152	1.472	0.1473	7.560	<0.001
LVEDA, cm^2^, (x̅±SD)	33.3 ± 4.3	33.7 ± 4.4	33.5 ± 4.2	33.2 ± 3.8	−0.391	0.698	−0.944	0.350	−1.545	0.129
RVEDA/LVEDA, (x̅±SD)	0.59 ± 0.07	0.59 ± 0.08	0.58 ± 0.07	0.62 ± 0.08	−1.485	0.144	2.623	0.012	6.491	<0.001
TAPSE, mm, (median [IQR])	16.0 [14.0, 18.0]	16.5 [15.0, 18.0]	17.0 [15.0, 18.0]	15.5 [14.0, 17.5]	1.842	0.065	−2.999	0.003	−4.707	<0.001
IVCmax, mm, (median [IQR])	19.8 [18.8, 23.2]	19.6 [17.8, 22.7]	19.8 [18.8, 23.1]	21.3 [20.1, 24.5]	2.843	0.005	5.674	<0.001	6.276	<0.001
IVCmin, mm, (median [IQR])	16.6 [14.3, 19.8]	16.2 [14.1, 18.9]	15.9 [13.9, 18.5]	17.8 [16.2, 20.0]	−0.841	0.401	4.277	<0.001	5.960	<0.001
DIVC, %, (median [IQR])	21.2 [16.5, 37.1]	20.5 [13.6, 32.6]	26.2 [17.6, 36.9]	23.1 [13.9, 31.3]	3.210	0.001	−0.310	0.757	−2.177	0.030
PASP, mmHg, (median [IQR])	35.0 [26.0, 43.5]	33.0 [23.5, 41.5]	34.0 [25.5, 43.0]	42.0 [35.0, 52.0]	0.883	0.377	5.717	<0.001	5.769	<0.001
LVEF, %, (x̅±SD)	55.3 ± 6.6	55.0 ± 6.2	55.4 ± 6.5	55.1 ± 7.1	0.955	0.344	0.175	0.862	−1.191	0.239
CVP, mmHg, (median [IQR])	12.0 [10.0, 13.0]	11.0 [9.5, 12.0]	12.0 [11.0, 13.0]	14.0 [13.0,15.0]	2.372	0.018	6.340	<0.001	6.304	<0.001
CI, L/min/m^2^, (median [IQR])	3.02 [2.58, 3.60]	3.07 [2.69, 3.67]	3.00 [2.73, 3.47]	2.63 [2.20, 2.95]	−1.858	0.063	−5.555	<0.001	−6.247	<0.001
SVI, ml/m^2^, (median [IQR])	25.8 [22.6, 31.2]	27.0 [24.2, 33.0]	27.3 [23.4, 32.2]	22.1 [18.4, 27.1]	−0.938	0.348	−5.054	<0.001	−5.929	<0.001
GEDVI, ml/m^2^, (median [IQR])	688.0 [653.0, 733.0]	689.0 [653.0, 734.5]	681.5 [645.0, 723.0]	679.7 [643.2, 715.2]	−3.954	<0.001	−2.788	0.005	−2.451	0.014
EVLWI, ml/kg, (median [IQR])	12.6 [11.0, 14.5]	13.1 [11.5, 14.8]	12.8 [11.0, 14.6]	12.4 [10.8, 14.6]	−5.336	<0.001	−5.154	<0.001	−2.663	0.008
PVPI, (median [IQR])	4.96 [4.30, 5.62]	5.13 [4.56, 5.66]	5.14 [4.33, 5.66]	5.02 [4.36, 5.58]	−0.719	0.472	−1.694	0.090	−0.710	0.478
Pplateau, cmH2O, (x̅±s)	26.4 ± 2.1	20.3 ± 2.2	25.1 ± 2.1	29.4 ± 1.9	29.936	<0.001	39.516	<0.001	22.937	<0.001
Driving Pressure, cmH2O, (x̅±s)	14.7 ± 2.5	15.3 ± 2.2	15.1 ± 2.1	14.4 ± 1.9	−1.704	0.095	−3.927	<0.001	−3.335	<0.001

ACP, acute cor pulmonale; RVEDA, right ventricular end-diastolic area; LVEDA, left ventricular end-diastolic area; TAPSE, tricuspid annular plane systolic excursion; IVC, inferior vena cava; DIVC, Distensibility index of the IVC; PASP, pulmonary artery systolic pressure; LVEF, left ventricular ejection fractions; CVP, central venous pressure; CI, cardiac index; SVI, stroke volume index; GEDVI, global end-diastolic volume index; EVLWI, extravascular lung water index; PVPI, pulmonary vascular permeability index.

### 3.3 Risk factors for the incidence of ACP in patients with moderate-to-severe ARDS

Upon conducting multivariable logistic regression analysis, incorporating potential variables identified through univariate analysis (*p* < 0.20), it was found that only PEEP >12 cmH_2_O emerged as an independent risk factor for ACP development in patients with moderate-to-severe ARDS (refer to Table 2 for details).

### 3.4 Effects of ACP on the prognosis of patients with moderate-to-severe ARDS

Before the intervention, patients with ACP showed higher incidence rates of AKI and ARF compared to those without ACP, although the differences were not statistically significant. There were no significant differences in the lengths of ICU stay and total hospital stay. However, among ICU survivors, those in the ACP group experienced longer ICU and total hospital stays compared to non-ACP patients, with statistically significant differences observed in total hospital stay (31.0 days vs. 20.7 days for non-ACP patients, *p* = 0.010). Regarding mortality risk, the ACP group exhibited higher rates of ICU mortality (46.2% vs. 10.3%, *p* = 0.010) and hospital mortality (53.8% vs. 10.3%, *p* = 0.003), as well as 28-day mortality (53.8% vs. 25.6%, *p* = 0.089) compared to the non-ACP group. The differences in ICU mortality and hospital mortality rates were statistically significant, whereas the difference in 28-day mortality did not reach statistical significance (refer to Table 4 for details).

**Table 4:** Prognosis of patients with moderate to severe ARDS with ACP.

Outcome	Total *n* = 52 (%)	ACP group *n* = 13 (%)	non-ACP group *n* = 39 (%)	z/χ^2^	*p*
AKI, *n* (%)	37 (71.2)	1 2(92.3)	25 (64.1)	_	0.078
ARF, *n* (%)	16 (30.8)	6 (46.2)	20 (51.2)	_	0.184
ICU stay, [median (IQR)], d	12.4 [6.5, 16.0]	12.5 [8.0, 12.0]	12.3 [6.0, 16.0]	0.360	0.719
Hospital stay, [median (IQR)], d	20.7 [12.0, 25.0]	23.8 [11.0, 29.0]	19.8 [12.0, 24.0]	−1.237	0.216
ICU stay in ICU survival group, [median (IQR)], d	13.0 [6.0, 16.0]	17.1 [10.0, 24.0]	12.2 [6.0, 16.0]	−1.066	0.286
Hospital stay in ICU survival group, [median (IQR)], d	22.5 [14.0, 26.0]	31.0 [25.0, 39.0]	20.7 [14.0, 24.0]	−2.567	0.010
ICU mortality, *n* (%)	10 (19.2)	6 (46.2)	4 (10.3)	_	0.010
Hospital mortality, *n* (%)	11 (21.2)	7 (53.8)	4 (10.3)	_	0.003
Day 28 mortality, *n* (%)	17 (32.7)	7 (53.8)	10 (25.6)	_	0.089

ACP, acute cor pulmonale; AKI, acute kidney injury; ARF, acute renal failure.

## 4 Discussion

ARDS is a prevalent clinical syndrome in the ICU (1, 2), which, in severe cases, can lead to increased pulmonary vascular resistance and pulmonary arterial hypertension. In 1977, WM. Zapol and MT. Snider first described pulmonary vascular dysfunction in ARDS patients, noting elevated pulmonary vascular resistance. This led to progressive right ventricular dysfunction and subsequent ACP, ultimately resulting in refractory circulatory failure after several days of respiratory support (12). In our study, the incidence of ARDS among critically ill patients in the authors’ hospital was 9.9%. The incidence of ACP in patients with moderate-to-severe ARDS was 25.0%, slightly higher than in previous studies (6, 11, 13). This could be attributed to a higher proportion of pneumonia patients included in our study population (67.3%), as pneumonia-induced ARDS has been identified as an independent risk factor for ACP (6, 11).

The development of ACP significantly worsens the prognosis of ARDS patients (6, 14), making the study of high-risk factors for ACP a hot topic in recent years. Boissier et al. found that pneumonia-induced ARDS and driving pressure were independent risk factors for ACP (6), while the study by Lhéritier et al. identified PaCO_2_ > 60 mmHg as the sole factor independently associated with ACP (13). A study by A. Mekontso Dessap et al., involving a large clinical cohort of 752 patients with moderate-to-severe ARDS, discovered four independent risk factors for ACP onset after limiting the tidal volume to 6–8 mL/kg and plateau pressure to ≤30 cmH2O. Specifically, these four factors were pneumonia-induced ARDS, a driving pressure > 18 cmH2O, a PaO_2_/FiO_2_ ratio < 150 mmHg, and a PaCO_2_ > 48 mmHg (11). In our study, the proportion of pneumonia cases in the ACP group was higher than that in the non-ACP group. Nevertheless, no statistically significant differences were observed, possibly due to sample size limitations. Although both driving pressure and PaCO_2_ are associated with mechanical ventilation settings, it is also possible that the severity of patient conditions in this study necessitated higher driving pressures, as well as imbalances in ventilation-perfusion ratio, elevated dead space ventilation, and decreased ventilation rate, resulting in increased PaCO_2_ levels. Hypercapnia itself acts as a potent vasoconstrictor for the pulmonary vasculature (15). Nevertheless, in our study, PaCO_2_ levels were excluded as an independent risk factor for ACP, possibly due to the small sample size. Additionally, multivariable logistic regression analysis identified PEEP >12 cmH_2_O as the sole independent risk factor for ACP occurrence. Inappropriate mechanical ventilation settings can lead to increased pulmonary vascular resistance and elevated pulmonary arterial pressure, leading to right ventricular dysfunction or even ACP, thereby prolonging mechanical ventilation time and worsening prognosis.

In a rat lung injury model, it was demonstrated that by reducing alveolar collapse, PEEP could promote the recruitment of collapsed alveoli, improve oxygenation, decrease pulmonary artery resistance, and mitigate right ventricular afterload, thereby preventing right ventricular dilation and acute right ventricular failure (16). For ARDS patients, insufficient PEEP can lead to persistent or cyclic alveolar collapse, resulting in refractory hypoxemia. Moreover, the shear forces generated by the cyclic collapse and reopening of some recruitable alveoli can further trigger or exacerbate lung injury. Conversely, excessive PEEP can cause alveolar overdistension, increase pulmonary vascular resistance, and impair pulmonary circulation and right ventricular function, ultimately leading to circulatory failure (7). In a randomized controlled trial involving 1,010 patients with moderate-to-severe ARDS across 120 ICUs in nine countries, Cavalcanti et al. found that compared to the low-PEEP approach, lung recruitment, and PEEP titration strategy (with consistently higher average PEEPs compared to the low PEEP strategy group) increased 28-day all-cause mortality (17). The potential reason might be high PEEP-induced alveolar overdistension and impaired hemodynamics. In this study, after controlling for other mechanical ventilation parameters, the incidence of ACP before the intervention (with PEEP at 11.5 ± 1.7 cmH2O) was 25.0%. Alternatively, during the trial, the incidence of ACP detected by echocardiography was 17.3, 21.2, and 38.5% under PEEPs of 5 cmH2O, 10 cmH2O, and 15 cmH2O, respectively. This indicates that changes in PEEPs can somewhat affect right ventricular function and the incidence of ACP.

In this study cohort, different PEEPs were analyzed for their impact on pressure indicators, echocardiographic parameters, hemodynamics, and oxygenation indices. It was found that indicators directly influenced by pressure transmission, such as PASP and CVP, exhibited a positive correlation with PEEP. Additionally, oxygenation showed a positive change, gradually improving with PEEP. Alternatively, among different volume-related indicators, LVEDA, GEDVI, and EVLWI decreased with increasing PEEP, while RVEDA did not experience a gradual change but only exhibited significant right ventricular dilation at high PEEP levels. When PEEP increased from 5 cmH2O to 10 cmH2O, there was no significant change in right ventricular systolic function. This is likely because the increased pressure restricted venous return and alleviated right ventricular preload, thereby decreasing the right ventricular end-diastolic volume. However, with PEEP further increasing to 15 cmH2O, right ventricular systolic function significantly decreased, leading to pronounced right ventricular dilation together with an increased afterload pressure. Meanwhile, TAPSE and SVI, indicators of right ventricular systolic function, initially increased and then decreased with increasing PEEPs, demonstrating optimal performance at moderate PEEP. This phenomenon aligns with the results of an animal study (18), where researchers found that in ARDS-induced beagle dogs receiving mechanical ventilation at various PEEPs, static lung compliance peaked at a PEEP of 6–8 cmH2O, and TAPSE and SVI increased within a certain PEEP range. However, with further PEEP increases, pulmonary vascular resistance escalated, and SVI declined. In our study, when PEEP was elevated to 15 cmH2O, TAPSE and SVI significantly decreased, yet left ventricular systolic function was not notably affected. In terms of cardiac output, CI showed a gradual decrease with increasing PEEP. This finding is similar to the findings of a previous study (19), where compared to low PEEPs, high PEEPs improved oxygenation and promoted alveolar recruitment but also led to hypercapnia and respiratory acidosis, as well as right ventricular dilation, left ventricular deformation, and a significant decrease in cardiac index. Thus, appropriate PEEP settings are crucial for maintaining oxygenation, right ventricular function, hemodynamic stability, and preventing ACP in ARDS patients. Nevertheless, further research is needed to determine how to titrate PEEP to balance oxygenation, extravascular lung water, right ventricular function, and circulatory stability. Theoretically, from a right ventricular function perspective, the ideal PEEP may requires maintaining right ventricular afterload at the lowest level, that is, ensuring minimal pulmonary vascular resistance and optimal lung compliance in ARDS patients. This necessitates dynamic monitoring of the patient’s respiratory mechanics, pulmonary artery pressure, and pulmonary vascular resistance.

ACP is a severe complication in ARDS patients. Its incidence is particularly high among those with severe ARDS, significantly worsening their prognosis (20). In this study, patients with ACP prior to the trial exhibited higher rates of concurrent AKI and ARF compared to those without ACP. This may be related to decreased cardiac output in the ACP group, leading to inadequate perfusion of vital organs. Alternatively, it may reflect more severe conditions, such as organ damage caused by inflammatory responses. Among ICU survivors, ACP patients required longer ICU and total hospital stays, indicating more challenging recovery compared to those without ACP. In terms of mortality, the ACP group showed higher short-term mortality rates than did the non-ACP group, although there was no statistically significant difference in 28-day mortality between the two groups. This suggests that ACP may essentially represent a severe stage of ARDS or the primary disease that is potentially transient and reversible. Once patients recover or move beyond this stage, their prognosis might depend more on the treatment of the underlying disease and comprehensive management, including nutrition and rehabilitation, than on the presence of ACP *per se*. Moreover, early studies, such as that by Lhéritier et al., found that ACP was not an independent risk factor for 28-day mortality in patients with moderate-to-severe ARDS (13). Nevertheless, more data generally support the notion that ACP increases mortality in ARDS patients (6, 11).

In summary, the incidence of ACP increased with increasing PEEP under mechanical ventilation. Compared to low PEEPs, high PEEPs enhanced oxygenation and reduced the extravascular lung water index. However, they also caused right ventricular dilation, significantly compromising right ventricular systolic function and decreasing CI. Appropriate PEEP settings can improve oxygenation and mitigate atelectasis, while optimizing cardiac output by affecting venous return and pulmonary vascular resistance. PEEP settings should be tailored to each patient’s condition to prevent the adverse effects of excessively high PEEP on cardiac output. Furthermore, critical care ultrasound tools and hemodynamic monitoring should be employed to dynamically assess right ventricular function and cardiac output in real-time, allowing for timely adaptation of the treatment plan (21).

This study has some limitations. Firstly, it is a single-center, paired cohort study with a patient population primarily consisting of severe pneumonia cases, which may not fully represent ARDS patients in other regions. Secondly, the trial utilized PEEPs of 5 cmH2O, 10 cmH2O, and 15 cmH2O, with substantial gaps between each level, and higher PEEPs were not investigated. Thirdly, pulmonary artery catheter monitoring was not performed on the participants. Additionally, ultrasound estimations of pulmonary artery pressure might entail notable inaccuracies, and the assessment of pulmonary vascular resistance, which significantly impacts the right ventricle, was unattainable. Therefore, further research is needed to confirm the relationship between PEEP and pulmonary vascular resistance. Fourthly, cardiac ultrasound measurements are highly operator dependent. Hence, measurements in this study were acquired by qualified ultrasound personnel to ensure data reliability and reproducibility.

## 5 Conclusion

The incidence of ACP in patients with moderate-to-severe ARDS increases with increasing PEEP. Compared to low PEEPs, high PEEPs can improve oxygenation and reduce extravascular lung water without significantly affecting the pulmonary vascular permeability index or left ventricular systolic function. However, it can also lead to right ventricular dilation and significantly reduces right ventricular systolic function and cardiac index, thereby inducing ACP.

## Data availability statement

The original contributions presented in the study are included in the article/supplementary material, further inquiries can be directed to the corresponding author.

## Ethics statement

The studies involving humans were approved by the Ethics Review Committee of Xiaolan People’s Hospital of ZhongShan. The studies were conducted in accordance with the local legislation and institutional requirements. The participants provided their written informed consent to participate in this study. The requirement of ethical approval was waived by the Ethics Review Committee of Xiaolan People’s Hospital of ZhongShan for the studies involving animals because No experiments were conducted on animals. The studies were conducted in accordance with the local legislation and institutional requirements.

## Author contributions

LX: Conceptualization, Writing – original draft, Writing – review & editing. GW: Project administration, Writing – original draft, Writing – review & editing. YQ: Data curation, Investigation, Writing – original draft, Writing – review & editing. ZH: Investigation, Writing – original draft, Writing – review & editing. ZZ: Investigation, Writing – original draft, Writing – review & editing. ZQ: Methodology, Writing – original draft, Writing – review & editing. YS: Formal Analysis, Writing – original draft, Writing – review & editing. ZF: Validation, Writing – original draft, Writing – review & editing. JZ: Supervision, Writing – original draft, Writing – review & editing.
